# Zeatin Regulates Somatic Embryogenesis in *Liriodendron sino-americanum* via *CYCD3*

**DOI:** 10.3390/plants14182823

**Published:** 2025-09-09

**Authors:** Yuanming Tang, Nannan Chen, Xiao Sun, Liming Zhu, Jinhui Chen, Ying Chen

**Affiliations:** State Key Laboratory of Tree Genetics and Breeding, Co-Innovation Center for Sustainable Forestry in Southern China, Nanjing Forestry University, Nanjing 210037, China; tangyuanming163@163.com (Y.T.); chennan@njfu.edu.cn (N.C.); sunxiao0075@163.com (X.S.); zhulm@njfu.edu.cn (L.Z.)

**Keywords:** *Liriodendron sino-americanum*, somatic embryogenesis, zeatin, *CYCD3*

## Abstract

Somatic embryogenesis (SE) is a crucial strategy for in vitro regeneration in woody plants, yet its efficiency is affected by multiple factors, and the underlying regulatory mechanisms remain insufficiently understood. In this study, callus tissues from two *Liriodendron sino-americanum* genotypes involving different hybrid combinations, ON-LoS and TN-LoS, were treated with varying concentrations (0, 0.005, 0.01, 0.05 mg/L) of exogenous zeatin (ZT) to evaluate its regulatory effect on SE. Treatment with 0.01 mg/L ZT significantly promoted SE in ON-LoS but suppressed it in TN-LoS, indicating that ZT elicited divergent regulatory effects on SE between the two genotypes. To explore the molecular basis of this divergence, transcriptome analysis was conducted at the early stage of SE. Differentially expressed genes (DEGs) were significantly enriched in hormone signaling, particularly in the cytokinin (CK) and brassinosteroid (BR) signaling pathways, as well as biosynthetic and redox-related pathways. In particular, given the established role of cell cycle-related gene *CYCD3* (*Lchi20922*) in promoting cell division, *CYCD3* was markedly upregulated by ZT in ON-LoS but downregulated in TN-LoS. These results indicate that ZT regulates SE efficiency through differential modulation of *CYCD3* expression in distinct genotypes. This study enhances our understanding of the molecular basis of SE regulation in *Liriodendron sino-americanum* and offers a theoretical framework for improving regeneration efficiency in woody plants.

## 1. Introduction

The genus *Liriodendron*, belonging to the family Magnoliaceae, includes only two species: the Chinese tulip tree (*L. chinense*) and the North American tulip tree (*L. tulipifera*) [1]. It holds significant phylogenetic importance among angiosperms and demonstrates relatively high resistance to both biotic and abiotic stresses, making it a valuable renewable biomaterial [2]. In modern cultivation, its excellent traits are inherited through asexual propagation techniques such as cuttings, grafting, and tissue culture.

Somatic embryogenesis (SE) provides a highly effective approach for clonal propagation and genetic transformation in *Liriodendron* species. SE refers to the process in which plant cells or tissues, under specific in vitro culture conditions, are induced to undergo a developmental pathway similar to zygotic embryogenesis, resulting in the formation of a new individual [3]. Despite successful SE induction in *Liriodendron*, the underlying molecular mechanisms, especially genotype-dependent regulation, remain poorly understood. It can also occur under natural conditions, where plant tissues or organs form structures resembling somatic embryos in response to environmental stress, such as in the leaves of *Bryophyllum pinnatum* [4]. The SE has been established in the *Liriodendron sino-americanum* [5,6]. Yet, some issues still need to be improved, such as increasing the efficiency of somatic embryo induction, reducing the number of abnormal embryos (Somatic embryos exhibiting irregular morphology), and the molecular regulatory network of *Liriodendron* SE are poorly understood. Despite the broad applicability of SE induction, its efficiency varies due to factors such as genotype, exogenous hormones, and other influencing elements.

Among these factors, genotype has been recognized as a key prerequisite for the successful induction of SE and plays a critical role in inducing embryogenic callus formation [7]. As an intrinsic factor, genotype influences SE frequency, which largely depends on genetic variation [8,9,10,11]. Experiments have demonstrated that SE can occur in all plants under suitable external conditions. Thus, controlling the culture conditions and understanding the influencing factors are critical during SE [12,13,14]. Different genotypes of explants may require distinct optimal culture conditions, leading to variations in SE frequency or even failure to induce somatic embryos [2]. A series of experiments on SE induction in the *Liriodendron sino-americanum* revealed significant differences in induction capacities among different hybrid combinations. The induction efficiencies for direct, reciprocal, and backcross hybrids were 2.43%, 5.98%, and 2.94%, respectively [15,16]. These findings indicate that the genotype significantly impacts SE induction, highlighting the influence of genetic factors. However, studies on how genotype affects SE remain limited, warranting further in-depth exploration.

Numerous studies have shown that the proper application of phytohormones is a major factor in the successful induction of SE in plants [17]. Cytokinins (CKs) play a pivotal role in SE by regulating cell proliferation, differentiation, and hormonal crosstalk during embryogenesis [18,19]. Zeatin (ZT) is a novel plant growth regulator belonging to the CK family. It stimulates plant cell division, accelerates metabolism and protein synthesis, promotes rapid plant growth, and enhances anti-aging and cold resistance abilities. ZT has been widely studied for its ability to enhance callus induction and SE efficiency [20]. ZT has been employed to induce SE in lychee [21]. Similarly, studies on *Phyllostachys edulis* (Moso bamboo), *Schisandra chinensis*, and *Gossypium* (cotton) have reported the application of ZT in SE, where it has been shown to promote the process [22,23,24]. In *Liriodendron sino-americanum*, ZT has been shown to induce SE [5]. Within a certain concentration range, ZT can enhance the efficiency of SE and promote normal somatic embryo development [25]. Nevertheless, the molecular mechanisms underlying ZT-regulated SE in *Liriodendron sino-americanum* remain largely unknown. With the widespread application of omics technologies in plant research, transcriptomics has been used for the identification and validation of key genes involved in somatic embryo development [26,27]. However, only a few transcriptomic studies have examined the molecular mechanisms regulated by CKs.

Although SE has been widely applied, the molecular mechanisms that initiate and regulate this process require further investigation [28]. We hypothesize that differences in SE efficiency among *Liriodendron* hybrids are due to genotype-dependent gene expression profiles influenced by ZT. In this study, we aim to utilize an established SE platform for the *Liriodendron sino-americanum* to investigate the molecular regulatory mechanisms affecting SE efficiency in hybrids with different genotypes. By applying exogenous plant hormones, the conditions for SE in the *Liriodendron sino-americanum* were meticulously optimized. Transcriptome sequencing was then performed to identify key differentially expressed genes (DEGs) during the early stages of SE in the *Liriodendron sino-americanums* with different genotypes. Functional enrichment analysis of DEGs, including Gene Ontology (GO) and Kyoto Encyclopedia of Genes and Genomes (KEGG) pathway analyses, was conducted to elucidate the critical signaling pathways involved in their regulation. The findings provide valuable insights for further studies on the molecular regulatory mechanisms underlying SE in the *Liriodendron sino-americanum*.

## 2. Results

### 2.1. Significant Differences in SE Efficiency of Different Liriodendron sino-americanum Genotypes Under ZT Treatment

**Figure 1:**
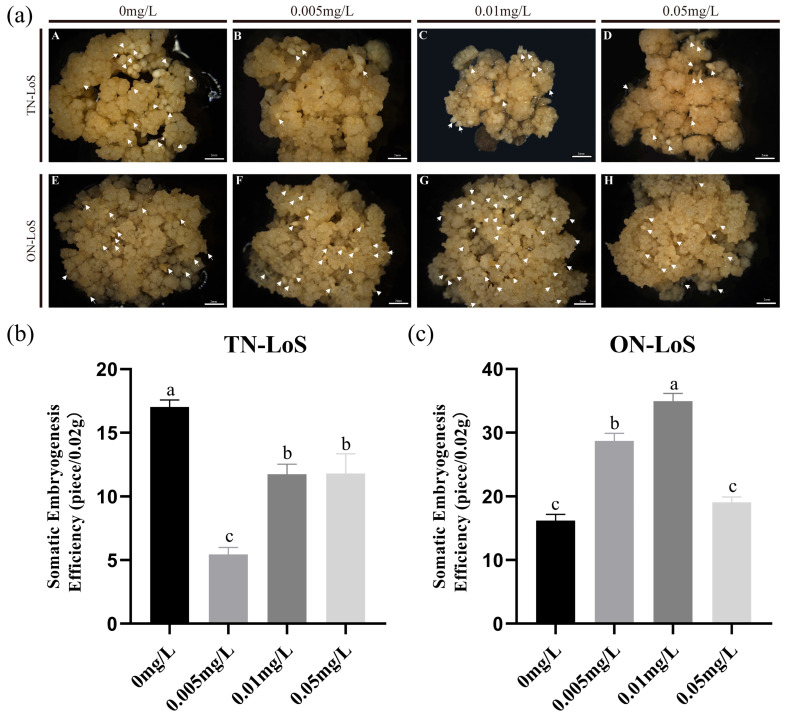
Analysis of SE Efficiency under Exogenous ZT Treatment. (**a**) Phenotypic images of SE in *Liriodendron sino-americanum* after 45 days of treatment with exogenous ZT (0, 0.005, 0.01, 0.05 mg/L) under different genotypes. (A–D) TN-LoS genotype; (E–H) ON-LoS genotype. Scale bar: 2 mm. (**b**,**c**) Statistical data of SE efficiency after 45 days of exogenous ZT treatment (0, 0.005, 0.01, 0.05 mg/L) under different genotypes. Data are presented as mean ± standard deviation (SD) of three biological replicates. Statistical analysis was performed using one-way ANOVA. Bars with the same letter indicate no significant difference (*p* > 0.05); bars with different letters indicate significant differences (*p* < 0.05); the white arrow indicates a somatic embryo (SE).

To investigate the effects of ZT on somatic embryo induction and morphological development in *Liriodendron sino-americanum*, we treated the embryogenic callus from ON-LoS and TN-LoS with ZT (Figure 1a). The results demonstrated that during somatic embryo induction, ZT had varying effects on the induction rate of somatic embryos in different genotypes. In ON-LoS, exogenous ZT significantly enhanced the somatic embryo induction rate and promoted normal embryonic development. (Normal embryo: Somatic embryos with typical morphology) The highest induction efficiency was observed at 0.01 mg/L ZT, which was significantly higher than that of the control group (*p* < 0.05) (Figure 1c). Conversely, in TN-LoS, exogenous ZT significantly suppressed somatic embryo induction. The highest induction rate (440 embryos/g) was recorded in the absence of exogenous ZT, which was significantly higher than that in ZT-treated groups (*p* < 0.05) (Figure 1b). Additionally, the proportion of abnormal embryos increased significantly. These results suggest that the differential response of genotypes to ZT treatment is closely associated with embryogenic competence. In ON-LoS, the high induction rate and normal embryo morphology under ZT treatment indicate strong embryogenic potential and support efficient downstream regeneration. In contrast, the suppressed SE efficiency and increased proportion of abnormal embryos in TN-LoS suggest compromised embryogenic competence. These abnormal embryos are often non-viable and fail to develop into healthy plantlets, ultimately reducing the regeneration quality and plantlet survival rate.

### 2.2. RNA-Seq and Transcript Annotation

To gain comprehensive insights into molecular regulatory mechanisms underlying the early-stage SE of the *Liriodendron sino-americanum* in response to exogenous ZT, we collected transcriptome sequencing on callus samples (referred to as stage CK) from two genotypes (ON-LoS and TN-LoS) after 21 days of proliferation, as well as samples subjected to 0 mg/L ZT treatment for 7 days (Stage 0) and 0.01 mg/L ZT treatment for 7 days (Stage 1). After discarding reads with linkers and those of low quality, we obtained 92.60 million clean reads (6.78 Gb in total), with a Q30 percentage exceeding 90.88% (Table S1). The mapping quality of the RNA-seq data is shown in Table S2. On average, 87.23% of the clean reads were successfully mapped to the *Liriodendron* reference genome, with a range from 85.93% to 88.55%. Among these, the unique mapping rate averaged 84.90%, and the multiple mapping rate was 2.33%, indicating that most reads mapped to a single location. Additionally, the properly paired mapping rate averaged 77.23% (range: 75.04–79.20%), reflecting good alignment integrity. These high-quality alignment results support the reliability of the data for downstream transcriptomic analyses.

**Figure 2:**
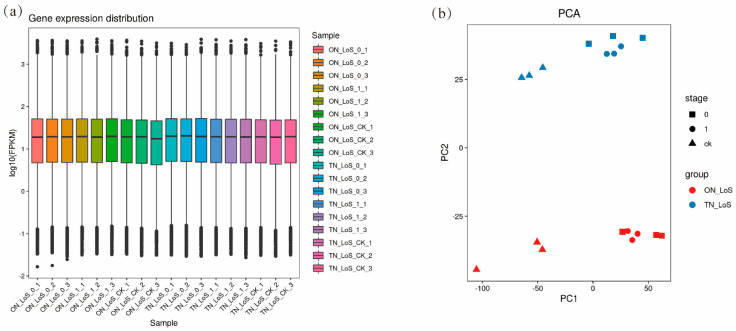
(**a**) Box plot depicting gene expression distributions for grouped samples. (**b**) Principal Component Analysis. Stage 0: SE in *Liriodendron sino-americanum* after 7 days of treatment with 0 mg/L exogenous ZT. Stage 1: SE in *Liriodendron sino-americanum* after 7 days of treatment with 0.01 mg/L exogenous ZT. Stage CK: the uninduced original callus samples of both genotypes without any ZT treatment.

Annotation was conducted using the *Liriodendron* reference genome, and the FPKM values for each sample are presented in Figure 2a. Principal component analysis (PCA) revealed a clear genetic divergence between the two *Liriodendron sino-americanum* genotypes, with four distinct clusters observed among the samples (Figure 2b), indicating significant genotype-dependent transcriptional differences. The three biological replicates within each treatment group clustered closely together, demonstrating high reproducibility and confirming the reliability of the RNA-Seq data for further analysis.

### 2.3. Transcriptomic Analysis of Differential Gene Expression During Early SE Induced by ZT in Liriodendron sino-americanum Genotypes

**Figure 3:**
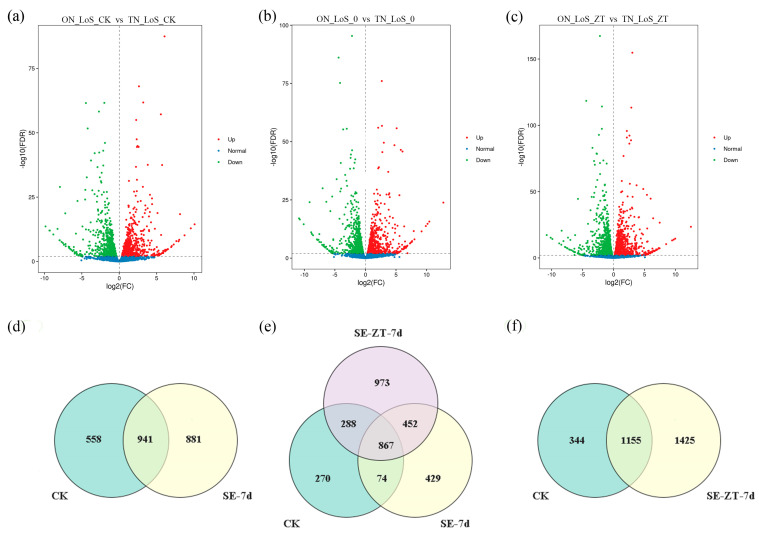
Volcano and Venn diagrams of DEGs under different genotypes and treatment conditions. Volcano plots of DEGs under different treatment conditions for *Liriodendron sino-americanum* with different genotypes: initial callus formation (**a**), without ZT application (**b**), and with ZT application (**c**). (**d**–**f**) Venn diagrams show the overlap of DEGs across ON-LoS and TN-LoS genotypes and time points. DEGs were identified using DESeq2 with |log_2_(fold change)| > 1 and adjusted *p*-value < 0.05 as thresholds.

To elucidate the molecular regulation of exogenous ZT during the early stage of SE in different *Liriodendron sino-americanum* genotypes, in this study, we conducted transcriptome sequencing on early-stage of SE samples from two genotypes (ON-LoS and TN-LoS). Comparative transcriptome analysis revealed a substantial number of DEGs (Figure 3). In the initial callus, the number of downregulated genes (4344) slightly exceeded that of upregulated genes (4193), which may reflect early repression of developmental or stress response genes to facilitate embryogenic transition. (Figure 3a). In the absence of ZT treatment, the number of upregulated genes (4545) was significantly higher than that of downregulated genes (4233), suggesting a general activation of gene expression associated with the onset of SE. (Figure 3b), whereas under ZT treatment, the numbers of upregulated (4115) and downregulated genes (4109) were nearly equal, indicating that ZT not only activates key genes promoting somatic embryogenesis but also represses genes that may inhibit this process or are associated with competing developmental pathways (Figure 3c).

Further analysis of DEGs at different developmental stages showed that before somatic embryo induction, a total of 1499 DEGs were identified between the ON-LoS and TN-LoS genotypes, accounting for 44.71% of the total genes. At the early stage without ZT induction (SE-7d), the number of DEGs increased to 1822 between the ON-LoS and TN-LoS genotypes, representing 54.34% of the total genes (Figure 3d,e). Following ZT induction (SE-ZT-7d), the number of DEGs significantly increased to 2580, comprising 76.95% of the total genes between the ON-LoS and TN-LoS genotypes (Figure 3e,f). Venn diagram analysis demonstrated distinct DEG expression patterns between the ON-LoS and TN-LoS genotypes under ZT treatment. Notably, ZT treatment markedly increased the number of DEGs, highlighting its strong regulatory impact on transcription during early SE.

### 2.4. Functional Enrichment Analysis of DEGs

**Figure 4:**
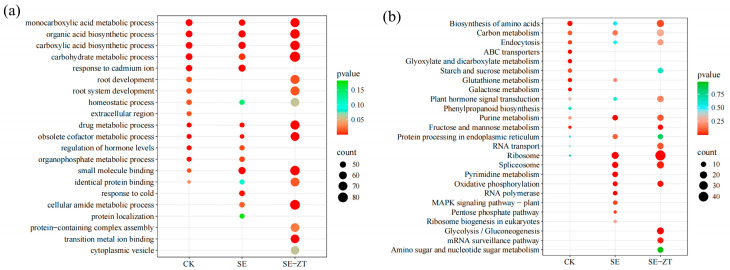
GO enrichment (**a**) and KEGG pathway enrichment (**b**) analysis of DEGs between the ON-LoS and TN-LoS genotypes under different treatments. The bubble size indicates the number of DEGs associated with each term (gene count), and the color gradient represents the enrichment significance based on −log_10_(*p*-value). Larger bubbles and darker colors suggest higher DEG counts and stronger statistical association, respectively. Terms are ranked by *p*-value, and the top categories are displayed.

To elucidate the molecular regulatory mechanisms underlying the effects of exogenous ZT on SE in the *Liriodendron sino-americanum*, we performed GO and KEGG enrichment analyses on DEGs in two genotypes (ON-LoS and TN-LoS) under different treatment conditions (Figure 4). GO analysis showed that DEGs were enriched in biological processes, cellular components, and molecular functions. Further analysis indicated that DEGs were predominantly associated with pathways related to small molecule binding, organic acid biosynthesis, and carbon metabolism. These genes function as oxidoreductases, transcription factors, and transporters, contributing to cellular metabolic regulation. To further investigate the key biological roles of DEGs, we performed KEGG enrichment analysis (Figure 4b). The results revealed that DEGs were predominantly enriched in pathways related to plant hormone signal transduction, carbon metabolism, glycolysis/gluconeogenesis, RNA transport, purine metabolism, oxidative phosphorylation, mRNA surveillance, amino sugar and nucleotide sugar metabolism, and starch and sucrose metabolism. During the early stages of SE in *Liriodendron sino-americanum* embryogenic callus, these pathways collectively orchestrate the regulation of embryogenesis initiation. Annotation of key genes within these pathways identified multiple genes involved in hormone signaling transduction.

### 2.5. Transcriptomic Changes in Phytohormone Signaling Pathways

**Figure 5:**
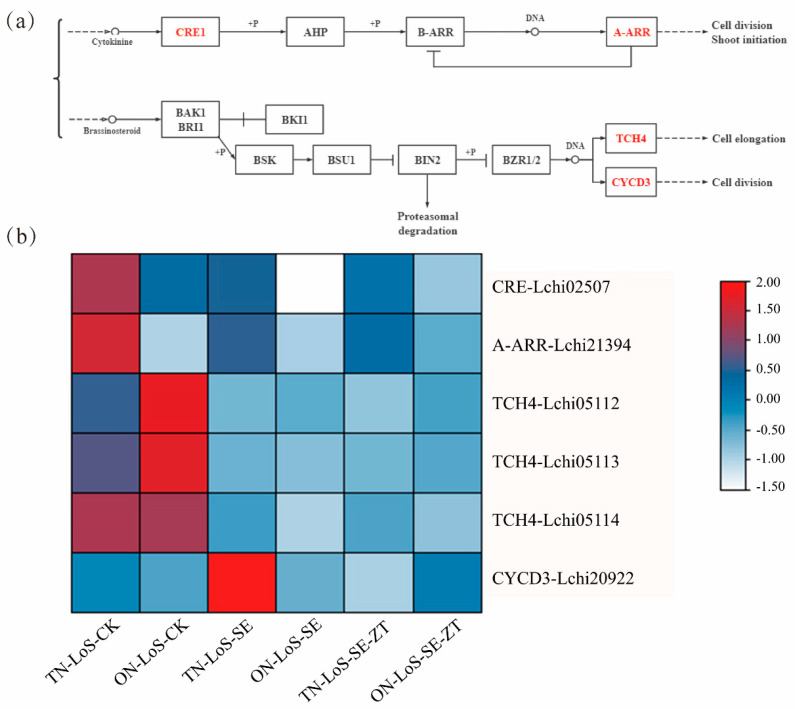
Visualization analysis of key pathways in plant hormone signal transduction. (**a**) Schematic diagram of CK and BR. (**b**) The heat map showed the expression profiles of genes related to CK and BR signal transduction. The color key on the right represents the expression changes in terms of Log_2_FC from upregulation (red) to downregulation (blue).

According to the KEGG functional enrichment analysis, we focused on the cytokinin (CK) and brassinosteroid (BR) signaling pathways to further investigate the molecular mechanisms underlying early SE in the ON-LoS and TN-LoS genotypes (Figure 5). DEGs in the cytokinin pathway included the cytokinin receptor gene *CRE* (*Lchi02507*) and the type-A response regulator *A-ARR* (*Lchi21394*). Their expression patterns differed significantly between the two genotypes during SE induction.

Notably, *A-ARR* was significantly downregulated in TN-LoS but upregulated in ON-LoS. Similarly, *CRE* expression was higher in TN-LoS under control conditions, but increased markedly in ON-LoS upon ZT treatment.

In the BR signaling pathway, three *TCH4* homologs (*Lchi05112*, *Lchi05113*, *Lchi05114*) and one *CYCD3* homolog (*Lchi20922*) showed differential expression. *TCH4* genes were generally downregulated in both genotypes, and *CYCD3* showed distinct expression patterns under ZT treatment, with significant upregulation in ON-LoS and downregulation in TN-LoS. Specifically, ZT significantly induced *CYCD3* in ON-LoS, where SE was enhanced, but repressed *CYCD3* in TN-LoS, where SE was inhibited. Given the established role of *CYCD3* in promoting cell division, these results suggest that ZT enhances SE efficiency in ON-LoS at least in part by activating cell cycle regulators.

Although the overall fold changes for some genes appear modest, the consistent expression trends across replicates and genotypes, coupled with the biological relevance of genes like *CYCD3*, support their involvement in hormone-mediated regulation of SE. Therefore, we propose that ZT modulates SE efficiency by regulating CK and BR signaling pathways, with *CYCD3* serving as a key downstream factor in this process.

### 2.6. Validation of DEGs via qRT-PCR

**Figure 6:**
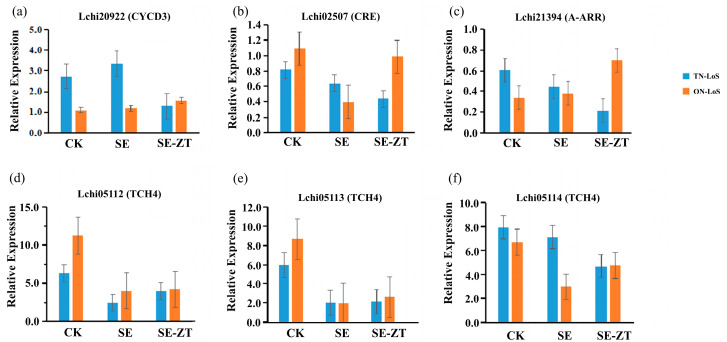
qRT-PCR validation of differentially expressed genes (DEGs) under different treatments and genotypes. The figure shows the relative expression levels of six genes under three treatment conditions: control (CK), somatic embryogenesis without ZT treatment (SE), and somatic embryogenesis with exogenous ZT treatment (SE + ZT). (**a**) Lchi02922 (CYCD3), (**b**) Lchi08207 (CRE), (**c**) Lchi21394 (A-ARR), (**d**) Lchi05112 (TCH4), (**e**) Lchi06133 (TCH4), (**f**) Lchi08114 (TCH4). Error bars represent the standard deviation (SD) of three biological replicates.

To validate the precision of the transcriptome data, quantitative real-time polymerase chain reaction (qRT-PCR) analysis was conducted on the candidate genes. The transcriptomic expression data (FPKM) corresponding to the selected genes have been provided in Table S3, and the Y-axis in Figure 6 represents relative expression levels from the qRT-PCR analysis. A comparison of the gene transcript abundances between the qRT-PCR and RNA-seq data yielded consistent outcomes (Figure 6), thereby corroborating the dependability of the transcriptome sequencing results. This further validates the regulatory function of key genes during the initial stages of SE in *Liriodendron sino-americanums* with diverse maternal genotypes.

### 2.7. Additional Transcriptomic Support from Other Datasets

**Figure 7:**
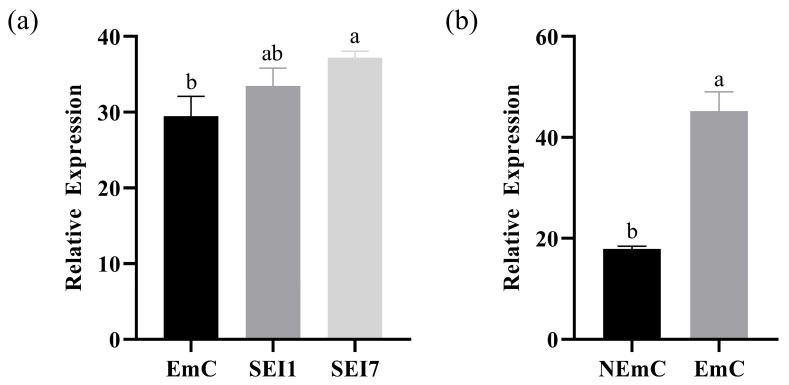
Expression pattern of *LcCYCD3* in various SE-related samples from *Liriodendron sino-americanum* based on independent transcriptome datasets. (**a**) Relative expression levels of *LcCYCD3* in embryogenic callus (EmC), somatic embryo induction day 1 (SEI1), and day 7 (SEI7). (**b**) Comparison of *LcCYCD3* expression between non-embryogenic callus (NEC) and embryogenic callus (EmC). Data are presented as mean ± SD of three biological replicates. Different letters indicate statistically significant differences (*p* < 0.05, one-way ANOVA followed by Duncan’s multiple range test).

To further support the potential regulatory role of *CYCD3* during (SE), we analyzed expression profiles of *LcCYCD3* from previously published transcriptome datasets of *Liriodendron sino-americanum* in our laboratory (Figure 7) [29]. These datasets included both embryogenic callus (EC) and non-embryogenic callus (NEC) samples, as well as samples across early SE stages (1 and 7 days after induction). (EC: Callus tissues that are capable of undergoing somatic embryogenesis and forming somatic embryos under appropriate induction conditions. NEC: Callus tissues that fail to initiate somatic embryogenesis.) In EC tissues, *LcCYCD3* exhibited significantly higher (*p* < 0.001) expression levels compared to NEC. Additionally, in the SE time-course dataset, *LcCYCD3* expression was gradually upregulated from the callus stage (EmC) to 1 day (SEI1) and 7 days (SEI7) after induction, consistent with a role in cell cycle activation during embryo initiation. These findings, although based on independent transcriptome datasets, provide additional evidence supporting the involvement of *CYCD3* in SE regulation, aligning with our main RNA-seq observations. Nevertheless, functional validation in the target genotype remains necessary in future studies.

## 3. Discussion

Exogenous CKs are commonly used to regulate SE [30]. Among the CKs, the most commonly used species is ZT. Studies on cotton have reported that the plant hormone ZT was used to induce somatic cell embryogenesis, and the induction rate was 33.3% [31]. Similarly, in a study on the regeneration system of *Jatropha curcas*, Galaz-Ávalos Rosa determined that cytokinin alone, as a growth regulator, was an effective approach for inducing SE from the leaves of *Jatropha curcas* [32]. In our research, ZT was applied during the SE process of different *Liriodendron sino-americanum* genotypes. The results showed that ZT had varying effects on different genotypes. Specifically, exogenous ZT treatment enhanced SE efficiency in the ON-LoS genotype but reduced it in the TN-LoS genotype. In *Litchi*, ZT has been reported to potentially increase the rate of abnormal embryo induction [30]. One possible explanation for the inhibitory effect of ZT on SE efficiency and *CYCD3* expression in the TN-LoS genotype is genotype-specific sensitivity to exogenous cytokinins. Different genotypes may have distinct receptor profiles or downstream signaling networks that respond differently to the same hormone treatment. In TN-LoS, it is plausible that higher sensitivity or altered signaling feedback mechanisms lead to an imbalance in cytokinin perception, causing suppression rather than promotion of key cell cycle regulators like *CYCD3*. This hypothesis aligns with the observed increase in abnormal embryos after ZT treatment in TN-LoS, suggesting that an excessive or misregulated cytokinin signal disrupts normal embryogenesis pathways in this genotype. Future work involving receptor expression profiling and hormone signaling pathway analyses will be crucial to confirm this genotype-dependent responsiveness. This is consistent with the significant increase in the proportion of abnormal embryos after ZT treatment in the TN-LoS genotype. Xing Denghui investigated changes in endogenous hormone levels during the SE of *Echinodorus crisis* L. Their results revealed that endogenous hormone levels continuously increased, with peaks for ZT and Zeatin riboside occurring at 15 days, followed by a gradual decline. Building on these findings, they examined the effects of various CKs on SE and observed that different types and concentrations of CKs exerted diverse induction effects. Among them, higher concentrations of ZT were more effective in inducing SE than lower concentrations [33]. Consistent with the findings of this study, it has been observed that CKs regulate SE in a similar manner across species, although variations exist. The primary factors contributing to these differences are likely complex molecular regulatory networks. ZT, as a hormone playing a crucial role in embryogenesis [34], has been shown to have its biosynthetic genes upregulated during the early SE process in *Garcinia mangostana*. Moreover, all 19 genes involved in the cytokinin pathway were upregulated, further suggesting that cytokinin-related genes may play a positive regulatory role in SE [35]. Therefore, to further enhance SE efficiency for forest tree genetic improvement, this complex molecular regulatory mechanism requires further investigation.

Plant hormone signaling plays a crucial role in the SE process [36,37]. We performed a comparative transcriptomic analysis during the early stage of SE. KEGG analysis revealed significant enrichment in the cytokinin and brassinosteroid signaling pathways. Cyclin D3 (CYCD3) is a class of cyclins that regulate the G1/S phase transition and play a critical role in plant growth and development. In recent years, studies have identified *CYCD3* as a key downstream target gene in the BR signaling pathway, where it plays a crucial role in SE [38]. Previous research has shown that during SE, BR upregulates *CYCD3* through the BZR1/BES1-dependent signaling pathway, thereby promoting cell cycle progression into the S phase and enhancing cell division capacity [39]. A study on Dimocarpus longan found that *cycd3* mutants led to apoptosis in embryogenic callus cells [40]. In our study, transcriptome and qRT-PCR results indicated that in the ON-LoS genotype, ZT treatment significantly upregulated *CYCD3* expression, coinciding with a higher SE induction rate. In contrast, in the TN-LoS genotype, ZT treatment suppressed *CYCD3* expression and significantly reduced SE induction efficiency. These findings align with the mechanism by which BR promotes *CYCD3*-mediated cell cycle progression, ultimately influencing SE induction. Nonetheless, the observed transcriptional trends of CYCD3 and related hormone signaling genes are consistent with the phenotypic trends in SE efficiency, and suggest a potential regulatory link between gene expression and embryogenic competence.

Additionally, *A-ARRs*, which are primary cytokinin response genes, function as negative regulators in the cytokinin signaling pathway by attenuating downstream transcriptional responses [41]. Suppression of *A-ARRs* has been shown to increase cytokinin sensitivity and promote SE in species such as *Arabidopsis* and *Medicago* [42,43]. In our study, ZT treatment suppressed *A-ARR* homolog expression in ON-LoS, consistent with enhanced cytokinin responsiveness and SE capacity. Conversely, sustained A-ARR expression in TN-LoS may have dampened the cytokinin signaling cascade, negatively affecting embryogenic induction.

Our findings suggest that the DEGs involved in hormone signal transduction may regulate the response of *Liriodendron sino-americanums* to ZT during SE. Exogenous ZT increased the SE efficiency of the ON-LoS genotype, whereas it inhibited SE in the TN-LoS genotype. Furthermore, the expression patterns of *CYCD3* homologs under both ZT-treated and untreated SE conditions in different *Liriodendron sino-americanums* suggest that the differential expression of *CYCD3* homologs may influence hormone synthesis, metabolism, and signal transduction, ultimately contributing to the observed differences in SE efficiency among *Liriodendron sino-americanums*.

## 4. Materials and Methods

### 4.1. Plant Materials and Zeatin Treatment

The experimental materials in this study were derived from *Liriodendron sino-americanum* trees produced through controlled pollination at the Anji Forestry Farm in Zhejiang in 2020. The genotypes involved different hybrid combinations, using Tennessee (TN) and Ontario (ON) from North America as the maternal parents, and Longshan (LoS) from eastern China as the paternal parent (Table 1). In early July 2020, aggregate fruits from different genotypes hybrid combinations were collected, sealed in plastic bags with proper labeling, and stored in ice boxes for transportation to the laboratory. Immature embryos were cultured on induction media to initiate the formation of embryogenic callus (3/4MS medium containing 2 mg/L 2,4-Dichlorophenoxyacetic acid (2,4-D) in a dark environment at 23 °C.), which was used as the starting material. Callus tissues, after 21 days of proliferation and with uniform growth status, were transferred to embryo induction media (EIM: 3/4MS medium without 2,4-D) with different concentrations of ZT (0, 0.005, 0.01, and 0.05 mg/L), and cultured at 23 °C in the dark. All genotypes and treatments were repeated 3 times in independent experiments. For each concentration, 9 Petri dishes were used, with 5 pieces of callus per dish, each piece weighing approximately 0.02 g, for a total of 0.1 g per dish. After 45 days, the total number of somatic embryos was counted for each genotype and treatment. Somatic embryogenesis (SE) efficiency was calculated as the number of embryos per gram of callus (embryos/g), representing the total number of embryos induced per gram of callus tissue.

### 4.2. RNA Extraction, Library Construction, and Sequencing

Samples stored at −80 °C were used for RNA extraction with the Eastep Super Total RNA Extraction Kit (manufactured by Promega Biotech Co., Ltd., Shanghai, China). RNA quality and DNA contamination were assessed by gel electrophoresis. RNA integrity was assessed with 1.2% agar gel electrophoresis, and RNA integrity and total amount were accurately detected with the Agilent 2100 bioanalyzer. The extracted RNA samples were preserved on dry ice and shipped to Novogene Co., Ltd. (Beijing, China) for RNA sequencing. According to the sequencing company’s protocol, TruSeq PE Cluster Kit v3-cBot-HS (Illumina, San Diego, CA, USA) was used to cluster the index-coded samples on the ACBot system. After cluster generation, libraries were prepared and sequenced on the Illumina NovaSeq 6000 platform (Illumina lnc., San Diego, CA, USA), producing 150 bp paired-end reads. Sequencing was performed by Novogene Co., Ltd. (Nanjing, China). For the proliferated callus stage (before ZT treatment), three biological replicates were sequenced for each genotype (ON-LoS and TN-LoS), totaling 6 samples. Under 0 mg/L ZT treatment (SE-7d), three biological replicates were sequenced for each genotype, totaling another 6 samples. Under 0.01 mg/L ZT treatment (SE-ZT-7d), three biological replicates were sequenced for each genotype, totaling 6 more samples. In total, 18 samples were sequenced (3 developmental stages × 2 genotypes × 3 replicates).

### 4.3. Data Processing and Analysis

Raw sequencing data were processed using in-house Python (v 3.8) scripts to remove reads containing adapter sequences or low-quality bases. The Q20, Q30, and GC content of the clean data were calculated using FastQC v0.11.9. Gene expression levels were quantified using featureCounts v1.6.4. [44]. The complete *Liriodendron* genome and annotation files were downloaded from an online database (https://ftp.cngb.org/pub/CNSA/data1/CNP0000295/CNS0044063/CNA0002404/ (accessed on 25 April 2025)) [45]. Clean reads were aligned to the reference genome using HISAT2 (v2.2.1). Transcript quantification was performed using HTSeq-count (v0.13.5) [46] with the corresponding GTF annotation file. Differentially expressed genes (DEGs) were identified using the DESeq2 (v1.16.1) package in R (v4.0.3) with a significance threshold of *p* < 0.01. Expression pattern clustering of DEGs was performed using the ggplot2 (v3.3.6) package, and Venn diagrams were created using TBtools (v2.323). GO (Gene Ontology) enrichment analysis of DEGs was conducted using the ClusterProfiler (v3.18.0) package, and KEGG pathway enrichment analysis was performed with the same package, using the full *Liriodendron* genome KEGG annotations as the enrichment background file (Org.db) [47]. Enrichment was conducted using the enricher function.

### 4.4. qRT-PCR Analysis

To validate the results obtained from RNA-seq data, qRT-PCR was performed to verify the DEGs. The primers for qRT-PCR were designed based on the RNA-seq-derived sequences from our study. Exclusion of primer-dimer or secondary structures verified using Primer-BLAST. First-strand cDNA was synthesized using the HiScript III 1st Strand cDNA Synthesis Kit (+gDNA wiper) (R312-01, Vazyme, Nanjing, China). Quantitative analysis of the transcriptional abundance of the candidate genes was carried out using the AceQ^®^ qPCR SYBR^®^ Green Master Mix (Q121-02) on a LightCycler 480 II system (Roche, Basel, Switzerland). Data were collected from three biological replicates. The *Liriodendron GAPDH* gene was used as a reference gene, 2^−ΔΔCT^ Method for Relative Gene Expression [48]. The primer information for qRT-PCR is provided in Table S4, the reaction components are shown in Table S5, and qRT-PCR process is shown in Table S6. Each sample included three biological replicates and three technical replicates.

### 4.5. Transcriptome Datasets Used for Expression Validation

To support the expression pattern of *LcCYCD3*, transcriptome datasets previously generated by our laboratory were used for additional analysis [29]. These datasets included samples from embryogenic callus (EmC), non-embryogenic callus (NEmC), and early somatic embryogenesis stages at 1 and 7 days after induction (SEI1 and SEI7), respectively. *LcCYCD3* expression data from these datasets were extracted and visualized using GraphPad Prism.

### 4.6. Statistical Analysis

All statistical analyses were conducted with GraphPad Prism software (version 5.0) and a significance level of *p* < 0.05 was deemed as a significant difference.

## 5. Conclusions

In summary, we have identified key genes that regulate SE capability by analyzing the differences in the responses of SE efficiency of *Liriodendron sino-americanum* to ZT among different genotypes. This progress has an important role for both the industrialization of *Liriodendron sino-americanum* cellular-engineering seedlings and the development of the forest cellular-engineering seed industry. These findings also lay a foundation for further research on the molecular regulatory mechanisms of SE in the *Liriodendron sino-americanum*. However, the molecular regulatory mechanisms of *CYCD3* homologous genes still necessitate further investigation, exploration, and validation. In-depth studies of these DEGs, including gene functional analysis through techniques like transgenics and gene editing, could offer functional validation and practical application for enhancing the SE efficiency in the *Liriodendron sino-americanum* of different genotypes.

## Figures and Tables

**Table 1:** Configuration of different genotype hybrid combinations.

Maternal Parents	Paternal Parent	Designation
Tennessee	Longshan	TN-LoS
Ontario	Longshan	ON-LoS

## Data Availability

The original contributions presented in this study are included in the article/Supplementary Materials, and further inquiries can be directed to the corresponding author.

## Supplementary Materials

The following supporting information can be downloaded at: https://www.mdpi.com/article/10.3390/plants14182823/s1, Table S1. Summary of transcriptome sequencing results after filtering. Table S2. Mapping percentage of RNA data. Table S3. DEGs expression from transcriptomic analysis. Table S4. Primer sequences for qRT-PCR. Table S5. Ingredient contents of qRT-PCR system. Table S6. qRT-PCR process.

## Abbreviations

The following abbreviations are used in this manuscript:

SE	Somatic Embryogenesis
ZT	Zeatin
CKs	Cytokinins
