# Supplementary material for: Zeatin Regulates Somatic Embryogenesis in Liriodendron sino-americanum via CYCD3
Source: Plants (Basel). 2025 Sep 9;14(18):2823. doi: 10.3390/plants14182823 (PMC12473157; doi:10.3390/plants14182823)
Supplement: Supplementary file 1 [file plants-14-02823-s001.zip › plants-3679908-supplementary.pdf]

Table S1. Summary of transcriptome sequencing results after filtering.

Sample	Raw Reads	Clean Reads	Clean Bases	Q20 Ratio	Q30 Ratio	GC Pct
TN-LoS-CK-1	43,740,070	43,659,416	6.55G	95.91	89.67	46.89
TN-LoS-CK-2	49,859,206	49,771,050	7.47G	95.93	89.70	47.03
TN-LoS-CK-3	44,907,558	44,821,324	6.72G	96.28	90.41	47.01
ON-LoS-CK-1	43,760,718	43,671,002	6.55G	96.14	90.11	47.21
ON-LoS-CK-2	47,238,860	47,148,930	7.07G	96.15	90.12	46.91
ON-LoS-CK-3	48,662,476	47,711,674	7.16G	96.07	89.97	47.29
TN-LoS-0-1	40,628,188	40,575,352	6.09G	96.89	91.67	46.62
TN-LoS-0-2	46,799,596	46,736,766	7.01G	96.88	91.58	46.35
TN-LoS-0-3	48,040,846	46,691,282	7.00G	96.96	91.78	46.59
ON-LoS-0-1	44,587,314	44,516,620	6.68G	96.50	90.85	47.10
ON-LoS-0-2	40,949,454	40,888,702	6.13G	96.68	91.22	47.28
ON-LoS-0-3	47,226,778	47,156,446	7.07G	96.83	91.60	47.13
TN-LoS-1-1	45,009,078	44,933,390	6.74G	96.23	90.32	47.36
TN-LoS-1-2	46,513,812	46,442,214	6.97G	96.59	91.09	47.34
TN-LoS-1-3	46,218,206	46,150,952	6.92G	96.73	91.38	47.37
ON-LoS-1-1	43,635,452	43,574,500	6.54G	96.81	91.51	47.01
ON-LoS-1-2	43,423,784	43,369,642	6.51G	96.80	91.43	47.05
ON-LoS-1-3	45,416,216	45,362,504	6.8G	96.79	91.42	46.59

Table S2. Mapping percentage of RNA data

Sample	Total reads	Unique Mapped(%)	Multiple Mappe(%)	Total Mapped(%)	Proper Map(%)
TN-LoS-CK-1	43,659,416	36,759,854(84.20)	963,680(2.21)	37,723,534(86.4)	33,324,896(76.33)
TN-LoS-CK-2	49,771,050	41,689,323(83.76)	1,101,215(2.21)	42,790,538(85.97)	37,568,976(75.48)
TN-LoS-CK-3	44,821,324	37,752,474(84.23)	1,008,036(2.25)	38,760,510(86.48)	34,323,042(76.58)
ON-LoS-CK-1	43,671,002	36,562,112(83.72)	966,417(2.21)	37,528,529(85.93)	32,768,618(75.04)
ON-LoS-CK-2	47,148,930	39,569,762(83.93)	1,052,224(2.23)	40,621,986(86.16)	35,641,646(75.59)
ON-LoS-CK-3	47,711,674	40,364,592(84.60)	1,033,081(2.17)	41,397,673(86.77)	37,106,684(77.77)
TN-LoS-0-1	40,575,352	34,793,505(85.75)	956,612(2.36)	35,750,117(88.11)	31,579,036(77.83)
TN-LoS-0-2	46,736,766	40,136,381(85.88)	1,118,837(2.39)	41,255,218(88.27)	36,545,310(78.19)
TN-LoS-0-3	46,691,282	40,196,411(86.09)	1,150,017(2.46)	41,346,428(88.55)	36,978,220(79.2)
ON-LoS-0-1	44,516,620	37,677,848(84.64)	1,045,060(2.35)	38,722,908(86.99)	34,369,642(77.21)
ON-LoS-0-2	40,888,702	34,733,351(84.95)	964,069(2.36)	35,697,420(87.30)	31,582,992(77.24)
ON-LoS-0-3	47,156,446	39,946,245(84.71)	1,135,102(2.41)	41,081,347(87.12)	36,321,232(77.02)
TN-LoS-1-1	44,933,390	38,098,650(84.79)	1,047,640(2.33)	39,146,290(87.12)	34,636,428(77.08)
TN-LoS-1-2	46,442,214	39,623,995(85.32)	1,092,074(2.35)	40,716,069(87.67)	36,355,454(78.28)
TN-LoS-1-3	46,150,952	39,568,104(85.74)	1,082,526(2.35)	40,650,630(88.08)	36,036,844(78.08)
ON-LoS-1-1	43,574,500	37,049,892(85.03)	1,053,204(2.42)	38,103,096(87.44)	33,727,168(77.40)
ON-LoS-1-2	43,369,642	37,094,245(85.53)	1,051,611(2.42)	38,145,856(87.96)	33,824,688(77.99)
ON-LoS-1-3	45,362,504	38,728,754(85.38)	1,104,191(2.43)	39,832,945(87.81)	35,273,720(77.76)

Table S3 DEGs expression from transcriptomic analysis

Sample	*CYCD3-Lchi20922*	*CRE-Lchi02507*	*A-ARR-Lchi21394*	*TCH4-Lchi05112*	*TCH4-Lchi05113*	*TCH4-Lchi05114*
ON-LoS-CK-1	2.989774451	9.160054258	2.339760101	126.1660836	87.98022296	76.06033447
ON-LoS-CK-2	2.003596712	10.16500748	2.39553987	105.8131098	97.58413956	68.79234833
ON-LoS-CK-3	4.580730193	9.435702149	4.38144932	104.7044304	67.02095181	65.80660749
TN-LoS-CK-1	3.907598424	14.06286385	14.43487511	73.14857801	58.55924809	74.4862456
TN-LoS-CK-2	4.514484815	10.03113043	10.45431321	75.71021651	54.30127228	61.37690418
TN-LoS-CK-3	3.253387358	11.55396924	11.94950951	73.71793001	63.57683911	77.98914055
ON-LoS-0-1	3.046747881	4.140350826	3.885602184	39.35195991	20.73658995	13.55426867
ON-LoS-0-2	2.849701768	6.638703036	2.855524418	35.23213216	19.52256674	14.02137136
ON-LoS-0-3	2.502173348	4.706455464	2.963156076	45.93450582	22.14141388	19.28790583
TN-LoS-0-1	9.456923523	12.41681331	10.74973403	30.19282063	25.26338052	25.49081177
TN-LoS-0-2	8.330014566	9.31474196	5.23586732	42.13132367	24.61004303	44.46994364
TN-LoS-0-3	7.414284081	8.10116951	9.885444319	35.70684851	24.91566987	23.79520893
ON-LoS-1-1	2.73384465	6.887740078	2.739430575	43.42754345	31.01967389	25.10913112
ON-LoS-1-2	4.107612675	5.768070349	8.103385925	34.3370747	21.16010206	17.46327619
ON-LoS-1-3	5.789483079	7.588574617	3.534648774	54.27187517	32.48928581	18.66739544
TN-LoS-1-1	1.64246409	9.752175105	9.909448008	27.43509587	26.31800087	29.3728051
TN-LoS-1-2	2.149934193	8.87311028	6.626187723	35.70928279	23.62719463	34.96718791
TN-LoS-1-3	1.914732749	9.32386548	6.63894784	28.05752226	20.80327633	24.11199633

The transcriptome sequencing data supporting the findings of this study have been deposited in the National Genomics Data Center (NGDC) under accession number: CRA027062 , and are publicly accessible at https://ngdc.cncb.ac.cn. The uploaded data will be made publicly available upon article publication.

Table S4 Primer sequences for qRT-PCR

Gene name		Primersequence
GAPDH	F	ACAACTAACTGCCTTGCTCCTT
	R	AGTCAGATCCACCACCGAAA
CYCD3-Lchi20922	F	ACACAGGAAGGCGACATAGAG
	R	TCAAAGCGGAACAAGGTCAGA
CRE-Lchi02507	F	TGGGGAGGGAAGGAAGATGAT
	R	CCCTCAAGGTCCAACGAAAGA
A-ARR-Lchi21394	F	TCTCACAGCAAGCACATTGGT
	R	ATCCGACAGCGTTCCCATTAC
TCH4-Lchi05112	F	CCGCTCCATCAACACTTTCCA
	R	CTTGCCTTCATTGCCTTGTGG
TCH4-Lchi05113	F	CTGCACTTCTTTGGCGTCTAC
	R	CATCGCATCCTTCCACTCCTT
TCH4-Lchi05114	F	TCCGTGAGGTCCGTGTAATCT
	R	TGAGCAGTAGTTGGTGAGTCG

Table S5 Ingredient contents of qRT-PCR system

Ingredient	Content
2×AceQ qPCR SYBR Green MasterMix	10.0 μL
Primer1	0.4 μL
Primer2	0.4 μL
cDNA	0.1-1.0 μM
ddH_2O up to20 uL	20 μL

Table S6 qRT-PCR process

Temperature	Cycles	Time
95°C	1	3 min
95°C		10 s
60°C	40	15 s
72°C		30 ~ 60 s/kb
72°C	1	5 min
4°C		∞
